# Adult-Onset Focal Bacterial Nephritis Without Urinary Symptoms Presenting With Systemic Toxic Rash: A Report of a Rare Case

**DOI:** 10.7759/cureus.96697

**Published:** 2025-11-12

**Authors:** Yoshihiro Kawaguchi, Toshiaki Kadokami, Kazuki Yamaguchi, Shuichiro Hayashi, Tsukasa Igawa

**Affiliations:** 1 Department of Urology, Saiseikai Futsukaichi Hospital, Fukuoka, JPN; 2 Department of Cardiology, Saiseikai Futsukaichi Hospital, Fukuoka, JPN; 3 Department of Dermatology, Saiseikai Futsukaichi Hospital, Fukuoka, JPN; 4 Department of Urology, Zeze Hospital, Oita, JPN; 5 Department of Urology, Kurume University School of Medicine, Kurume, JPN

**Keywords:** atopic dermatitis, bloodstream infection, focal bacterial nephritis, staphylococcus aureus, toxic rash

## Abstract

Focal bacterial nephritis (FBN) is a localized bacterial infection of the kidney, representing an intermediate stage between acute pyelonephritis and renal abscess. It is usually accompanied by urinary symptoms, and adult cases without such symptoms are rare. We report a 31-year-old woman with a history of atopic dermatitis who presented with high fever and a generalized toxic rash but no urinary tract symptoms. Laboratory data showed leukocytosis, markedly elevated inflammatory markers, and disseminated intravascular coagulation. Urinalysis was normal, but blood cultures were positive for methicillin-sensitive *Staphylococcus aureus* (MSSA). Contrast-enhanced computed tomography revealed multiple wedge-shaped perfusion defects in the right kidney, consistent with FBN. Echocardiography excluded endocarditis or embolic sources. The patient was treated with cefepime followed by a four-week course of cefazolin, resulting in complete clinical recovery and radiological improvement. No recurrence was observed during follow-up. This case illustrates that MSSA bacteremia can lead to FBN even in the absence of urinary symptoms. Early recognition and prompt imaging are crucial for diagnosis. Furthermore, systemic toxic rash may serve as an early clinical clue to underlying bacteremia.

## Introduction

Focal bacterial nephritis (FBN), also known as acute lobar nephronia, is a localized bacterial infection of the renal parenchyma that represents an intermediate stage between acute pyelonephritis and renal abscess formation. It typically presents with fever, flank pain, and pyuria and is most often caused by ascending urinary tract infection, although hematogenous spread can also occur [1,2].

FBN is typically seen in children and is considered a pre-renal abscess stage requiring long-term treatment [1,2]. While rare in adults, it usually presents with urinary tract symptoms, and diabetes mellitus is a known risk factor [3]. In a recent adult cohort (n=238), the median age was approximately 47 years, 86% had fever, and 56% had lower urinary tract symptoms, suggesting that adult FBN may be under-recognized [1]. Predictive markers such as elevated procalcitonin or urinary tract malformations have been proposed to improve early detection [4].

We report a case of FBN in an adult who initially presented with an infectious toxic rash, notably without any signs of a urinary tract infection.

## Case presentation

The patient was a 31-year-old woman with a history of atopic dermatitis, for which she had been prescribed betamethasone, tacrolimus, and Propet ointment topically for many years. She had no history of diabetes mellitus.

She initially presented to another hospital with a fever and a raised rash on her extremities and trunk, transferring to our hospital the following day. On admission, her vital signs were as follows: temperature 39.4°C, pulse rate 107 beats/min, and blood pressure 79/53 mmHg. By the time of her visit to our clinic, her skin symptoms had improved, and she reported no obvious lumbar back pain.

Initial lab results showed no abnormalities in her urine leukocyte count. Blood tests revealed a white blood cell (WBC) count of 20.8×10^3^/µl with 97% neutrophils, a creatinine (Cre) level of 1.11 mg/dl, a C-reactive protein (CRP) level of 23.44 mg/dl, a D-dimer level of 109.6 μg/ml, an international normalized ratio (INR) of 1.91, and a procalcitonin concentration of 11.65 ng/ml, indicating a severe infection (Table 1).

**Table 1:** Initial laboratory findings All abnormal values indicate severe systemic infection. WBC: white blood cell; Cre: creatinine; CRP: C-reactive protein; INR: international normalized ratio

Parameter	Result	Reference range	Units
Urine leukocyte count	Normal	0-5	/HPF
WBC	20.8	3.3-8.6	×10^3^/μl
Neutrophils (%)	97	37-72	%
Cre	1.11	0.46-0.79	mg/dl
CRP	23.44	0-0.14	mg/dl
D-dimer	109.6	<1.0	μg/ml
INR	1.91	0.8-1.2	-
Procalcitonin	11.65	0-0.5	ng/ml

**Figure 1:**
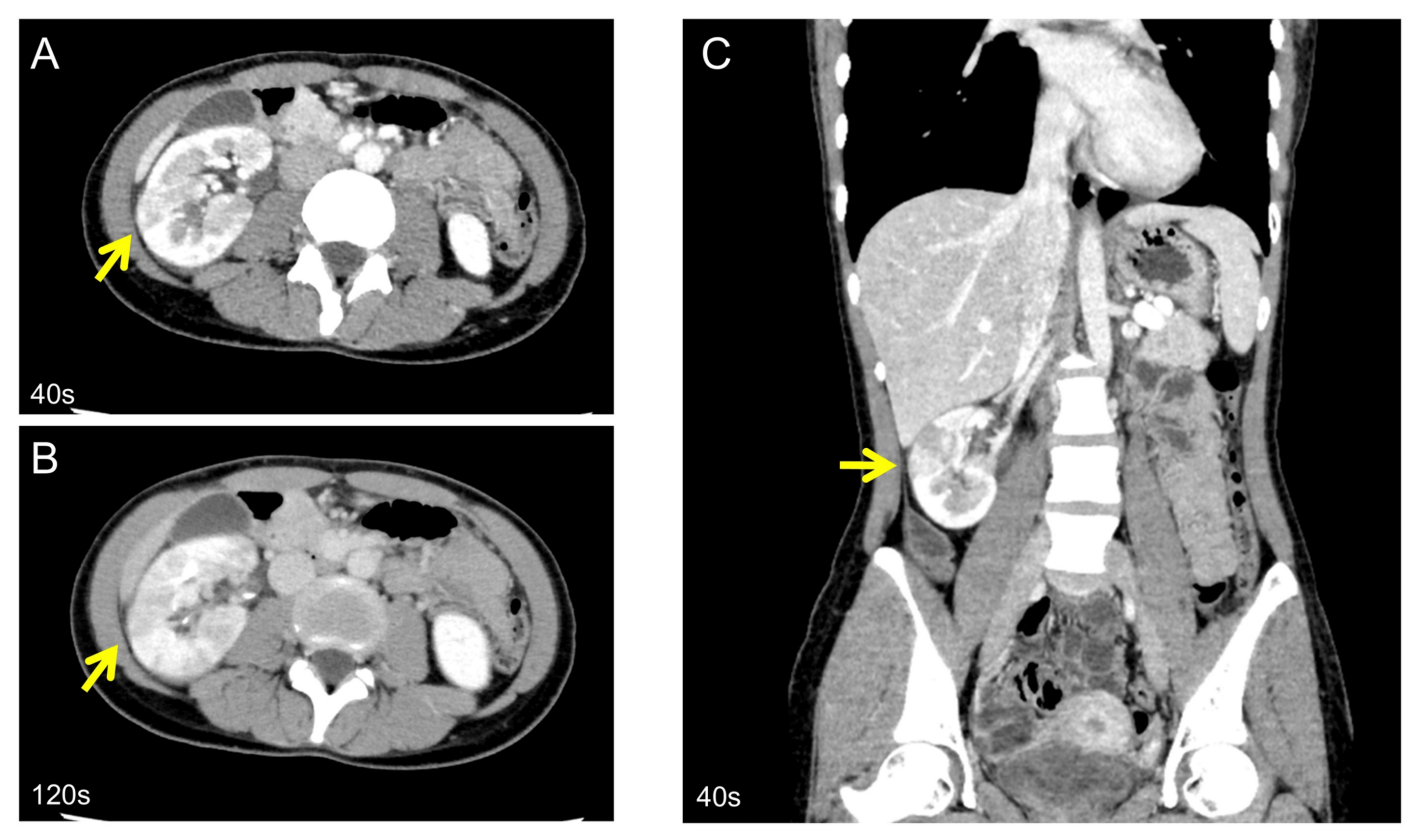
CT findings of focal renal perfusion defects Contrast-enhanced CT shows wedge-shaped to patchy areas of perfusion defect (A-C). These defects were most clearly seen in the delayed phase (B). Yellow arrows indicate the areas of perfusion defect. CT: computed tomography

After confirming she was not pregnant, a contrast-enhanced computed tomography (CT) scan was performed. The CT revealed an enlarged right kidney with multiple wedge-shaped areas of poor contrast (Figure 1) and clear contrast between the early and late contrast layers.

Treatment began with heparin for one day, thrombomodulin alpha for three days, and cefepime. While her urine culture was negative, blood cultures were positive for methicillin-sensitive *Staphylococcus aureus *(MSSA). Further investigations, including echocardiography, head CT, and lumbar magnetic resonance imaging (MRI), revealed no other infectious lesions.

**Figure 2:**
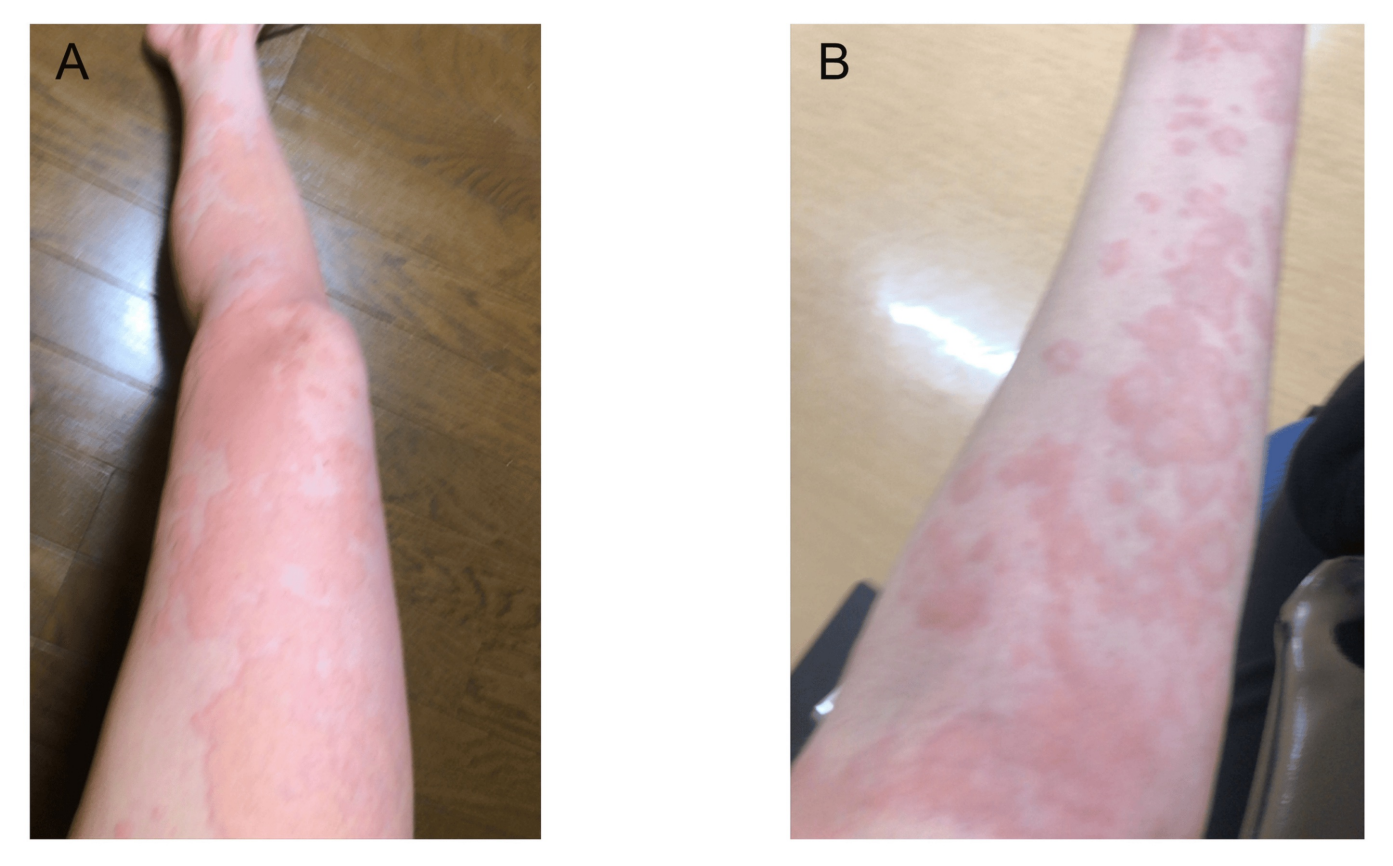
Skin manifestations of infectious toxicosis (A) Wheals with erythema on the lower limbs. (B) Wheals with erythema on the forearms. Dermatological consultation was performed, and infectious toxicosis was confirmed.

The patient was diagnosed with focal bacterial nephritis due to MSSA bacteremia and started on long-term antimicrobial therapy. She also presented with redness on her extremities, leading to a dermatologist's consultation and a diagnosis of infectious toxic rash (Figure 2).

**Figure 3:**
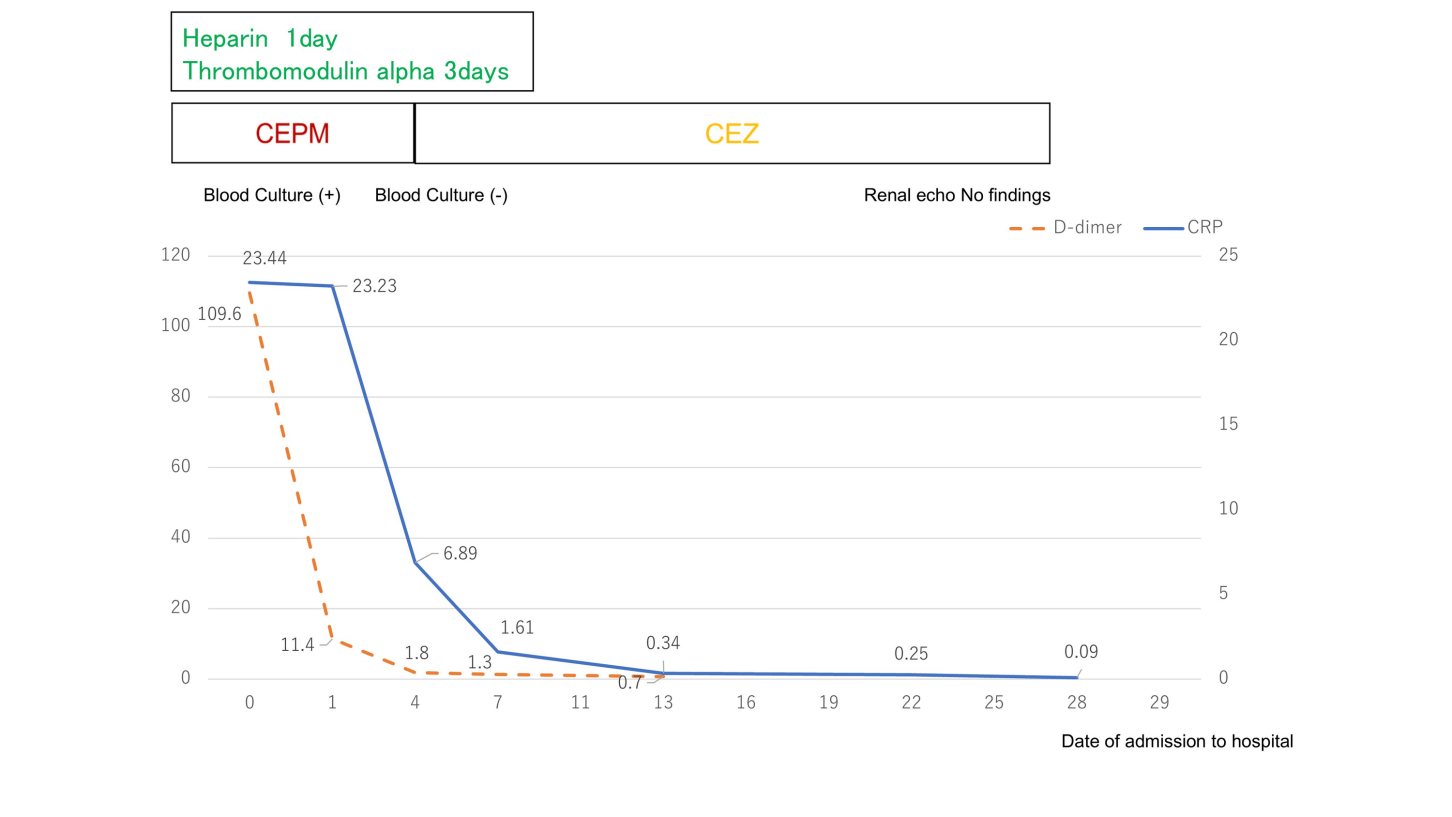
Graph of the treatment course Both inflammation and D-dimer levels showed rapid improvement. CEZ: cefazolin; CEPM: cefepime hydrochloride; CRP: C-reactive protein

On day 4 of hospitalization, follow-up blood cultures were negative. The patient received a long-term infusion of cefazolin (CEZ), which improved her blood sample levels. She was discharged on day 28 after the absence of echographic abnormalities was confirmed. She completed 28 days of antibiotic treatment and remained symptom-free during the outpatient follow-up (Figure 3).

## Discussion

This case highlights two crucial findings. Firstly, MSSA bacteremia can cause FBN even without urinary symptoms, and contrast-enhanced CT is instrumental in its diagnosis. Secondly, early systemic signs, such as an infectious toxic rash, should prompt immediate suspicion and treatment for bacteremia.

While FBN is typically considered secondary to urinary tract findings, in this case, MSSA was detected in blood cultures despite the absence of urinary tract symptoms. Although some reports indicate FBN can occur without urinary findings [5], and urine culture positivity rates are generally high (e.g., 233/238 or 97.9%), negative cases are rarely documented [1]. This suggests that hematogenous infection of the renal parenchyma is the predominant cause in such instances, making diagnosis difficult based on urinary findings alone. Other potential infection sites, such as the heart, bones, and eyes, were ruled out by echocardiography, head CT, and lumbar spine MRI, respectively, in this patient [6]. Therefore, contrast-enhanced CT was essential for diagnosis. Radiologically, FBN can mimic renal infarction due to its wedge-shaped hypoenhancing areas on contrast-enhanced CT. However, renal infarction usually presents with a well-defined cortical rim sign, whereas FBN tends to show patchy or striated hypoenhancement with indistinct borders. In this case, the lesion showed no cortical rim sign and responded favorably to antibiotic therapy alone, consistent with FBN rather than renal infarction. Echocardiography also ruled out cardiac vegetations or embolic sources. FBN can indeed occur in adults, and contrast-enhanced CT proves effective in these cases [1,7]. When FBN presents with atypical symptoms, long-term antibiotic therapy is recommended, as seen in an autopsy case where four weeks of treatment were administered despite no urinary tract symptoms.

Early systemic signs, such as an infectious toxic rash, are critical indicators for the detection and prompt treatment of bacteremia. In the present case, a bulging rash appeared on the extremities concurrently with the fever. It is plausible that the patient's underlying atopic dermatitis compromised her skin barrier function, facilitating MSSA invasion. A cross-sectional prospective study found skin lesions in 9% of bacteremia cases, with gram-positive bacteria like *Staphylococcus aureus *and *Streptococcus *being the most frequently detected organisms [8]. Staphylococcal sepsis has also been observed in children with atopic dermatitis [9]. Some studies propose that atopic dermatitis provides a favorable, yet underreported, environment for *Staphylococcus aureus *colonization, growth, and invasion [10]. However, despite these reports, a clear causal relationship between atopic dermatitis and MSSA bacteremia cannot be definitively established in this specific case. Furthermore, it remains unclear why the infection was confined to the right kidney, especially given the severity of the disseminated intravascular coagulation (DIC). A hematogenous infection, possibly secondary to an initial renal parenchyma infection, could be considered. Given the high D-dimer level, a pathology like renal infarction due to septic embolism warrants consideration.

This case emphasizes the importance of a thorough whole-body examination for deep-seated infections, including bacteremia and FBN, even in young women presenting with cutaneous findings and nonspecific systemic symptoms.

## Conclusions

This case emphasizes that MSSA bacteremia can cause acute bacterial nephritis without urinary findings and that contrast-enhanced CT is essential for diagnosis. Furthermore, early generalized skin eruptions serve as crucial clinical clues to underlying bacteremia and should expedite the initiation of treatment.

## References

[1] Jiao S, Yan Z, Zhang C, Li J, Zhu J (2022). Clinical features of acute focal bacterial nephritis in adults. Sci Rep.

[2] Campos-Franco J, Macia C, Huelga E, Diaz-Louzao C, Gude F, Alende R, Gonzalez-Quintela A (2017). Acute focal bacterial nephritis in a cohort of hospitalized adult patients with acute pyelonephritis. Assessment of risk factors and a predictive model. Eur J Intern Med.

[3] Sieger N, Kyriazis I, Schaudinn A (2017). Acute focal bacterial nephritis is associated with invasive diagnostic procedures - a cohort of 138 cases extracted through a systematic review. BMC Infect Dis.

[4] Lucas García J, Oltra Benavent M, Ferrando Monleón S, Marín Sierra J, Rabasco Álvarez MD, Benito Julve P (2020). Predictive markers of acute focal bacterial nephritis. A multicentre case-control study [Article in Spanish]. An Pediatr (Engl Ed).

[5] Horigome A, Uryu H, Takasago S, Atsumi Y, Mochizuki S (2024). Acute focal bacterial nephritis and bacteremia due to Staphylococcus simulans following an upper respiratory infection in a child: a case report. Cureus.

[6] Patel D, Jahnke MN (2015). Serious complications from Staphylococcal aureus in atopic dermatitis. Pediatr Dermatol.

[7] Guella A, Khan A, Jarrah D (2019). Acute focal bacterial nephritis: two cases and review of the literature. Can J Kidney Health Dis.

[8] Taquin H, Hubiche T, Roudière L, Fribourg A, Del Giudice P (2019). Prevalence and clinical characteristics of cutaneous manifestations associated with bacteraemia: a cross-sectional prospective study. Acta Derm Venereol.

[9] Hoeger PH, Ganschow R, Finger G (2000). Staphylococcal septicemia in children with atopic dermatitis. Pediatr Dermatol.

[10] Benenson S, Zimhony O, Dahan D, Solomon M, Raveh D, Schlesinger Y, Yinnon AM (2005). Atopic dermatitis-a risk factor for invasive Staphylococcus aureus infections: two cases and review. Am J Med.

